# Importance of a C-Terminal Conserved Region of Chk1 for Checkpoint Function

**DOI:** 10.1371/journal.pone.0001427

**Published:** 2008-01-09

**Authors:** Carmela Palermo, Justin C. Hope, Greg A. Freyer, Hui Rao, Nancy C. Walworth

**Affiliations:** 1 Department of Pharmacology, University of Medicine and Dentistry, New Jersey (UMDNJ), Robert Wood Johnson Medical School, Piscataway, New Jersey, United States of America; 2 Joint Graduate Program in Cellular and Molecular Pharmacology, University of Medicine and Dentistry, New Jersey (UMDNJ), Graduate School of Biomedical Sciences and Rutgers, State University of New Jersey, Piscataway, New Jersey, United States of America; 3 Graduate Programs in Environmental Health Sciences and Anatomy and Cell Biology, Columbia University, New York, New York, United States of America; 4 The Cancer Institute of New Jersey, New Brunswick, New Jersey, United States of America; Fred Hutchinson Cancer Research Center, United States of America

## Abstract

**Background:**

The protein kinase Chk1 is an essential component of the DNA damage checkpoint pathway. Chk1 is phosphorylated and activated in the fission yeast *Schizosaccharomyces pombe* when cells are exposed to agents that damage DNA. Phosphorylation, kinase activation, and nuclear accumulation are events critical to the ability of Chk1 to induce a transient delay in cell cycle progression. The catalytic domain of Chk1 is well-conserved amongst all species, while there are only a few regions of homology within the C-terminus. A potential pseudosubstrate domain exists in the C-terminus of *S. pombe* Chk1, raising the possibility that the C-terminus acts to inhibit the catalytic domain through interaction of this domain with the substrate binding site.

**Methodology/Principal Findings:**

To evaluate this hypothesis, we characterized mutations in the pseudosubstrate region. Mutation of a conserved aspartic acid at position 469 to alanine or glycine compromises Chk1 function when the mutants are integrated as single copies, demonstrating that this domain of Chk1 is critical for function. Our data does not support, however, the hypothesis that the domain acts to inhibit Chk1 function as other mutations in the amino acids predicted to comprise the pseudosubstrate do not result in constitutive activation of the protein. When expressed in multi-copy, Chk1D469A remains non-functional. In contrast, multi-copy Chk1D469G confers cell survival and imposes a checkpoint delay in response to some, though not all forms of DNA damage.

**Conclusions/Significance:**

Thus, we conclude that this C-terminal region of Chk1 is important for checkpoint function and predict that a limiting factor capable of associating with Chk1D469G, but not Chk1D469A, interacts with Chk1 to elicit checkpoint activation in response to a subset of DNA lesions.

## Introduction

The DNA damage checkpoint pathway consists of DNA damage sensors, signal transducers, and effectors that interface with the cell cycle machinery to prevent inappropriate transitions when DNA repair is required. In *Schizosaccharomyces pombe* the protein kinase Chk1 is an integral component of the checkpoint pathway, responsible for transducing a delay signal to the cell cycle machinery [1], [2]. Chk1 is essential for survival in response to DNA damage, as fission yeast cells lacking the *chk1* gene do not delay cell cycle progression, resulting in hypersensitivity to DNA damaging agents [1], [3]. Likewise, mammalian cells treated with inhibitors of Chk1 activity are sensitized to killing by DNA damaging agents [4], [5]. Genetic and biochemical dissection of the checkpoint pathway in *S. pombe* has uncovered at least eight additional components that lie at its core (Rad1, Rad3, Rad9, Rad17, Rad26, Hus1, Cut5, and Crb2), all of which are essential for Chk1 activation [2], [3], [6]–[9] Rad24, a 14-3-3 protein identified as a checkpoint mutant is also critical for Chk1 function as it binds to phosphorylated Chk1 and facilitates its nuclear localization following DNA damage [10], [11].

The precise mechanism by which Chk1 is activated in response to various DNA lesions has yet to be elucidated. Structurally, Chk1 consists of a highly conserved N-terminal kinase domain and a C-terminal non-catalytic domain. Evidence is mounting that critical aspects of Chk1 function are mediated through the C-terminal domain. We have previously reported that serine at position 345 in fission yeast Chk1 is phosphorylated *in vivo* and is required to activate Chk1 kinase activity in the presence of DNA damage [12]. Human Chk1 is also phosphorylated at S345, emphasizing the evolutionary importance of Chk1 in the checkpoint pathway [13], [14]. Phosphorylation at these SQ sites in mammalian cells and fission yeast is achieved via ATR and its homolog Rad3, respectively, and is also dependent on the aforementioned checkpoint proteins [1], [2], [14]–[16]. We and others have demonstrated that phosphorylation of S345 is critical for an intact checkpoint response, as mutations of this site that prevent phosphorylation compromise cellular survival in response to DNA damage [12], [16].

Chk1 itself is a serine/threonine kinase that can phosphorylate the Wee1 tyrosine kinase and the Cdc25 tyrosine phosphatase *in vitro* [17], [18]. Wee1 and Cdc25 regulate the activity of the cyclin-dependent kinase Cdc2 that controls entry into mitosis [19]. Hence, activated Chk1 achieves a cell cycle delay by targeting Wee1 and Cdc25. We have previously reported that Chk1 possesses a basal level of kinase activity that is elevated at least two-fold in response to DNA damage [12]. The fact that Chk1 retains a basal level of kinase activity in the absence of checkpoint induction may explain why overexpression of Chk1 leads to a sustained G2 arrest, which is independent of Chk1 phosphorylation and the checkpoint pathway, and from which cells cannot recover. While mutating the phosphoacceptor site (S345A) does not affect Chk1 basal activity, phosphorylation is critical for Chk1's increased kinase activity towards substrates in the presence of damage [12]. Thus, the C-terminus plays a crucial role in regulating Chk1 catalytic activity in response to DNA damage.

Studies of *Xenopus* and mammalian Chk1 support a model whereby the C-terminus structurally impinges on the N-terminal domain to suppress Chk1 catalytic activity in the absence of phosphorylation [20], [21]. Specifically, analysis of the Chk1 N-terminal kinase domain crystal structure reveals that in the absence of the C-terminal domain, it retains an open conformation and is 20-fold more catalytically active towards substrates [21]. Many kinases require phosphorylation within a region of the catalytic domain, known as the activation loop that serves as a molecular switch to turn on catalytic activity. Phosphorylation of a site within this loop secures the conformation of the activation and catalytic loops by stabilizing a cluster of positively charged side chains. There is no evidence that Chk1 is phosphorylated within this portion of the catalytic domain [13]. Indeed, Chk1 appears to have substitutions of specific residues in the activation loop in lieu of phosphorylation, which consequently creates a basic environment to stabilize the activated structure [21].

The observation that Chk1 has enhanced catalytic activity in the absence of the C-terminal half of the protein strongly suggests that this region possesses an autoinhibitory domain. An investigation of Xenopus Chk1 demonstrated the presence of a sequence in the C-terminal domain that could serve to inactivate the protein in a cell free extract system [22]. A study focusing on a liver-specific isoform of Chk1 in rat that consists of only the C-terminal non-catalytic domain demonstrated that this fragment binds to the kinase domain of the Chk1 protein, consistent with the possibility that it could act to regulate the protein's activity [23]. In addition, a short region of amino acids in the C-terminus of Chk1 resembles the Chk1 substrate recognition site [24], [25]. To test the hypothesis that this region in the C-terminal domain might act as a pseudosubstrate to confer autoinhibition, mutations were made at this site and the phenotypes of cells expressing the mutant proteins analyzed. The data obtained do not support a role for this domain as an autoinhibitory site. However, the conserved aspartic acid residue at position 469 instead emerged as an important site for Chk1 activity. Specifically, substitution of aspartic acid 469 with glycine confers partial function when expressed in multiple copies, whereas substitution with alanine exhibits a loss of function phenotype even in multiple copies. Further investigation of the D469G mutant revealed that it only functions in multiple copies when cells are exposed to forms of DNA damage that result in base modifications or strand breaks. The mutant is non-functional when cells experience the collapse of replication forks or contain unligated DNA. This data is consistent with the possibility that substitution of D469 with glycine allows flexibility in the conformation of Chk1 that is not permitted when D469 is substituted with alanine. As a result, the conformational flexibility conferred by the glycine substitution permits Chk1 to respond to some, but not all forms of DNA damage.

## Results

### A putative pseudosubstrate domain in the C-terminus of Chk1

**Figure 1:**
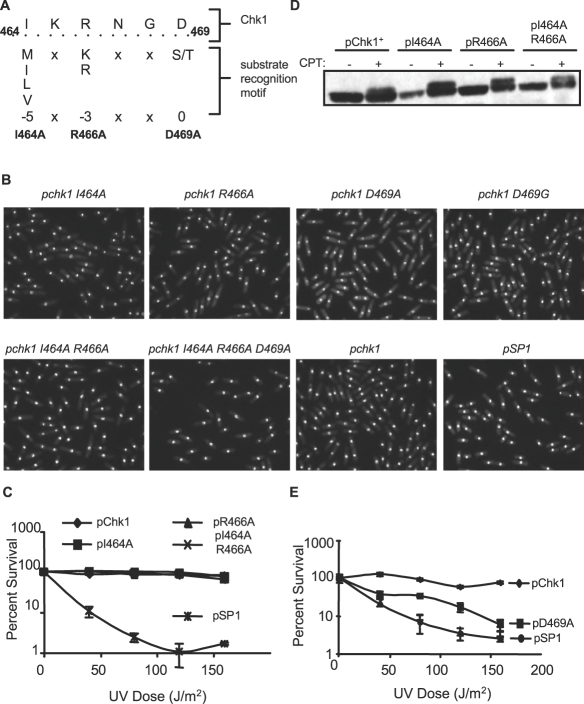
Mutation of D469 to alanine in a putative pseudosubstrate domain of Chk1 compromises function. A. Comparison of the region of fission yeast Chk1 that matches the Chk1 substrate recognition motif [24]. Mutations to alanine were generated at positions 464, 466 and 469 as indicated. B. The *chk1Δ* strains expressing the indicated plasmids, as well as the pSP1 empty vector as a control, were grown to mid-log phase, ethanol fixed, and DAPI stained for microscopic analysis. Expression of the mutant plasmids does not affect cellular length or morphology as compared to cells expressing the *wt chk1.* C. The *chk1Δ* strains expressing the indicated plasmids were grown to mid-log phase, spread on PMA plates in triplicate, exposed to 0, 40, 80, 120, or 160 J/m^2^ UV, and incubated for 3 days at 30°C. Mean survival was calculated as the percentage of colonies appearing on untreated plates. The data shown is from one experiment that is representative of 3 independent experiments. Error bars represent the standard deviation of the mean. D. Mutations at I464 and R466 do not compromise Chk1 phosphorylation upon exposure to UV light. Cycling cells were exposed to 40 µM CPT for 2 hours then collected and processed for Western blot analysis with antibody to the HA epitope at the C-terminus of each of the Chk1 proteins. E. Mutation of D469 to alanine compromises function. Cells were assayed as in B.

Using an in vitro kinase assay and variations of a known Chk1 substrate peptide, Hutchins et al. identified a sequence motif for Chk1 substrate recognition consisting of a hydrophobic residue (M, I, L, or V) at position –5 and a basic residue (R or K) at position –3, relative to the phosphoacceptor site [24] (Fig. 1A). A similar consensus was determined independently by different investigators [25]. Intriguingly, the sequence context of a region within the C-terminal domain of Chk1 spanning residues 464-469 matches this Chk1 substrate recognition motif, with a hydrophobic residue (I464) and a basic residue (R466) at positions –5 and –3 relative to aspartic acid at position 469, occupying the location that would be substituted with serine in a true substrate. Given the observation that the isolated kinase domain of Chk1 is more active than the full-length protein [21], we hypothesized that this region of Chk1 could act as a pseudosubstrate, permitting the C-terminal domain to autoinhibit the catalytic domain.

To test this hypothesis, mutations to alanine were made at positions I464, R466 and D469 as well as the double mutant I464A,R466A and the triple mutant I464A,R466A,D469A. If these residues bind the catalytic domain to maintain Chk1 in an inactive state in the absence of damage, the autoinhibition model predicts that alanine substitutions at these sites would disrupt such intra-domain interactions and render Chk1 constitutively active. Hence, substitutions in the predicted pseudosubstrate domain should result in an elongated cellular morphology indicative of a sustained checkpoint delay in the absence of damage. Plasmids expressing these mutants were introduced into a strain with a deletion of the *chk1* gene and the resulting strains were evaluated microscopically for an elongated phenotype. Contrary to what would be expected for constitutively active mutants, the individual alanine substitutions for I464, R466 and D469 (as well as the double and triple mutants) did not result in an elongated cellular phenotype (Fig. 1B). In fact, in the absence of damage, all of the mutants resemble a control strain transformed with the wild type plasmid suggesting that mutation of these residues does not affect cell cycle progression in the absence of damage. The mutants were also tested for Chk1 checkpoint function as measured by the ability to survive exposure to DNA damage generated by UV light. As shown in Fig. 1C, the I464A and R466A mutants, as well as the double mutant, are as functional as wild type Chk1. Furthermore, each of these three mutated proteins is phosphorylated in response to DNA damage generated by the topoisomerase I poison camptothecin (CPT, Fig. 1D). In contrast, substitution of alanine for D469 compromises the function of Chk1, rendering cells sensitive to UV light (Fig. 1E). Thus, while mutating residues within the hypothetical pseudosubstrate site had no effect on Chk1 activation in the absence of damage, arguing against the pseudosubstrate hypothesis, mutation of D469 did compromise function in the presence of DNA damage indicating that the highly conserved aspartic acid at that position is important for Chk1 function in the DNA damage checkpoint pathway.

### Allele specific behavior of mutations at D469

**Figure 2:**
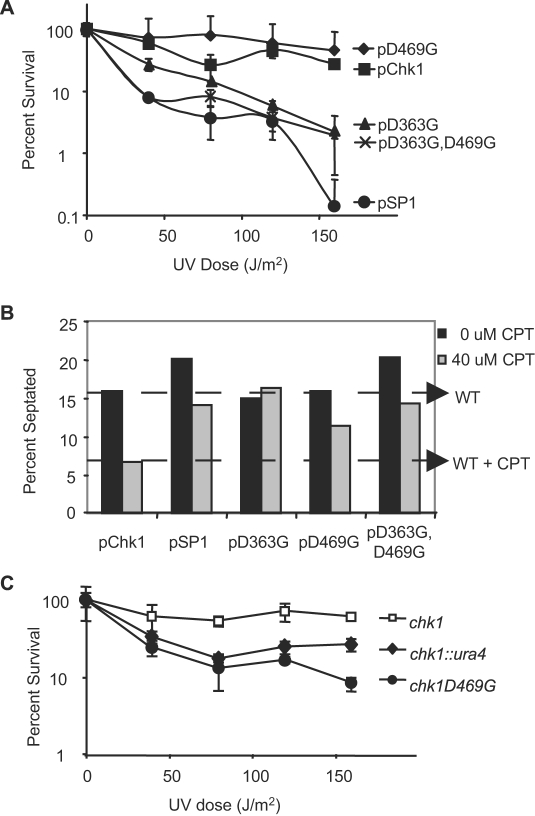
When expressed in multiple copies Chk1D469G does not compromise survival in response to UV light or CPT treatment. A. The *chk1Δ* strains transformed with the indicated plasmids were assayed for UV survival as described in Fig. 1B. B. The *chk1Δ* strains transformed with the indicated plasmids were grown to mid-log phase than treated or not with 40 µM CPT for 2 hours. Cells were collected and fixed for staining with calcofluor to monitor septation index. Graphed data is representative of three independent experiments. A total of 300 cells were counted for each strain and condition tested. C. A strain with *chk1D469G* integrated at the genomic *chk1* locus does not survive exposure to UV light. The indicated strains were assayed for UV sensitivity as described in Fig. 1B except that cells were grown and plated on rich media (YEA).

In a previous genetic screen searching for damage-sensitive alleles of *chk1*, we identified a mutant with a substitution of glycine for aspartic acid at position 469 along with substitution of glycine for aspartic acid at a non-conserved position, 363 [26]. To determine whether the two mutations contribute independently to Chk1 function, single mutants were constructed to produce Chk1D363G and Chk1D469G from their own promoters on multi-copy plasmids. As shown in Fig. 2A, expression of either the Chk1D363G,D469G or the Chk1D363G protein fails to restore function to the checkpoint deficient *chk1::ura4* strain, suggesting that D363 is important for function of the fission yeast protein despite the fact that it is not a well-conserved amino acid [27]. On the other hand, and to our surprise given the behavior of the Chk1D469A mutant (Fig. 1E), expression of Chk1D469G fully restores Chk1 function (Fig. 2A). To determine whether this response was unique to damage caused by UV light, we exposed cells to CPT and assayed for a checkpoint response as measured by a decrease in septation index (SI) in the presence of drug. As shown in Fig. 2B, *chk1::ura4* cells expressing the wild-type protein (pChk1) exhibit a decrease in SI whereas cells with an empty vector (pSP1) do not. While cells expressing either pD363G or pD363G,D469G do not exhibit a drop in SI, indicative of a non-functional checkpoint, cells expressing the D469G protein are responsive to CPT (Fig. 2B).

Given the behavior of the Chk1D469G allele expressed on a multi-copy plasmid, we sought to determine whether the mutant protein could function when integrated in single copy in the chromosome. As shown in Fig. 2C, cells with an integrated allele of *chk1D469G* behave much like cells with a disrupted copy of *chk1* demonstrating that only in multiple copies is the Chk1D469G protein capable of function. The lack of function when present in single copy is consistent with the possibility that Chk1D469G targets, responds to or interacts with a factor that is limiting for the DNA damage response.

**Figure 3:**
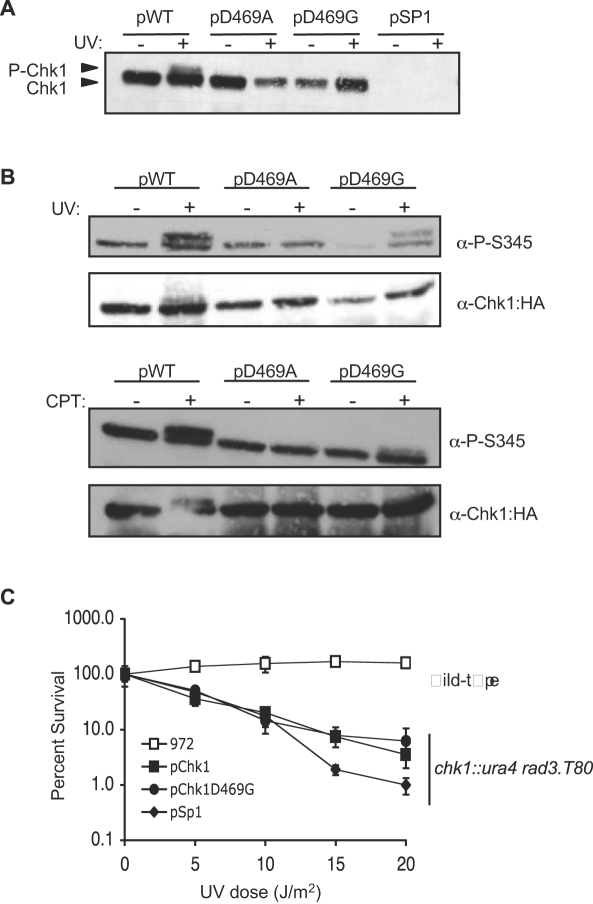
Chk1D469G exhibits a low level of phosphorylation in response to UV-irradiation or CPT treatment. A. Chk1D469G fails to display a mobility shift when whole cell lysates from UV irradiated cells are analyzed by Western blot. The *chk1Δ* strains expressing WT Chk1, Chk1D469A, Chk1D469G, or the empty vector (pSP1) were grown to mid-log phase, irradiated at a dose of 100 J/m^2^, and harvested for lysate preparation. Lysates were separated by SDS-PAGE and examined by Western blot analysis for the HA tag on Chk1. B. The *chk1Δ* strains expressing WT Chk1, Chk1D469A, or ChklD469G were grown to mid-log phase and either treated with 100 J/m^2^ UV (upper panels) or 40 µM CPT (lower panels). Lysates were prepared and subject to immunoprecipitation with the F7 anti-HA antibody. The immunoprecipitates were separated by SDS-PAGE and Western blots were probed with either the S345-phosphopeptide antibody or the Y11 anti-HA antibody. C. Rad3 activity is required for Chk1D469G survival in response to UV damage. The WT strain (972) and the *chk1Δ rad3.T80* strains expressing WT Chk1, Chk1D469G, or the empty vector control (pSP1) were grown to mid-log phase, spread on PMA plates, exposed to 0, 5, 10, 15, or 20 J/m^2^ UV, and incubated at 30°C. Survival was determined as described for Fig. 1B.

The observation that multiple copies of Chk1D469G confer a checkpoint response whereas multiple copies of Chk1D469A do not, led us to consider the basis for this allele specific response and examine the behavior of the two mutant proteins more closely. Chk1 is phosphorylated in response to DNA damage and most, but not all mutations that compromise Chk1 phosphorylation also compromise checkpoint function [12], [26], [28]. Phosphorylation can usually be detected as a mobility shift by SDS-PAGE when epitope tagged versions of Chk1 are detected with antibody to the tag. As shown in Fig. 3A, it is possible to detect phosphorylated wild-type Chk1 when the protein is expressed from the pSP1 plasmid, though the proportion of shifted Chk1 is less than that observed when protein produced from integrated alleles of *chk1* is examined (see, for example, Walworth and Bernards, 1996). Surprisingly, by Western blot of a whole cell extract, it was not possible to detect phosphorylation of Chk1D469G upon exposure to UV light, despite the robust behavior of the protein in the UV survival assay. Given the generally strong correlation between phosphorylation and function, we examined Chk1 phosphorylation more carefully by immunoprecipitation with the HA antibody followed by Western blot with an antibody raised against a phosphorylated serine 345-containing peptide. As shown in Fig. 3B, we were able to detect a mobility shifted, P-345 reactive form of the Chk1D469G protein in cells exposed to either UV light (top panel) or CPT (bottom panel). Consistent with the observation that Chk1D469A does not function in response to DNA damage, phosphorylation of this protein was not detected. We conclude that Chk1D469G is phosphorylated in response to DNA damage, albeit to a lesser extent than the wild-type protein. Nonetheless, despite the lower level of phosphorylation relative to the wild-type protein, Chk1D469G functions well in survival assays.

Phosphorylation of Chk1 normally requires the protein kinase Rad3 [2], [15] as well as several additional components of the checkpoint pathway that lie upstream of Chk1 and are required for both Chk1 phosphorylation and initiation of a mitotic delay [2], [6]. To determine if Chk1D469G, with its low level of phosphorylation, requires Rad3-dependent activation, we transformed the plasmid expressing the mutant protein into a strain containing a loss of function mutation of *rad3* and analyzed the ability of the strain to survive UV damage. A wild-type strain (972) with its checkpoint pathway intact is quite resistant to UV damage; however, loss of Rad3 function severely compromises survival, even if Chk1 is proficient (Fig. 3C). Similar to a strain expressing WT Chk1, UV irradiation bears lethal consequences on the *chk1D469G* strain when Rad3 function is deficient (Fig. 3C). Thus, survival of a strain expressing Chk1D469G still requires activation of the checkpoint pathway by Rad3.

**Figure 4:**
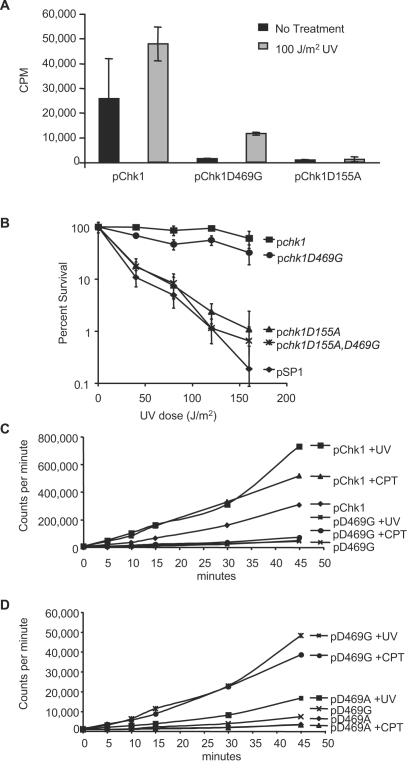
Chk1D469G has catalytic activity that increases in response to DNA damage. A. Chk1D469G has a reduced level of basal activity that increases in response to UV-irradiation. Chk1, Chk1D469G, and Chk1D155A immunoprecipitates from either untreated or UV-irradiated cells were assayed for their ability to phosphorylate a peptide substrate. The data shown depicts values for a single experiment with triplicate samples. It is representative of 3 independent experiments. B. The *chk1Δ* strains expressing the indicated plasmids were assayed for sensitivity to UV light as described in Fig. 1B. C. Chk1 and Chk1D469G immunoprecipitates from either untreated, UV-irradiated, or CPT-treated cells were assayed for their ability to phosphorylate a peptide substrate over the course of 45 minutes. The reduced level of Chk1D469G catalytic activity is not due to a delay in the onset of activation. D. Chk1D469A and Chk1D469G immunoprecipitates from either untreated, UV-irradiated, or CPT-treated cells were assayed for their ability to phosphorylate a peptide substrate over the course of 45 minutes. Chk1D469G displays higher levels of catalytic activity that increases in response to DNA damage compared to Chk1D469A. The data shown in C and D is representative of 3 independent experiments.

To further characterize the Chk1D469G allele we examined its catalytic activity using a peptide-based assay. As shown in Fig. 4A, Chk1D469G immunoprecipitated from untreated cells has essentially background levels of kinase activity, approximately the level of signal observed when the “kinase dead” Chk1D155A protein is assayed. Yet, activity of Chk1D469G, like that of wild-type Chk1, is stimulated several fold when the kinase is immunoprecipitated from cells exposed to UV light. Given the limited level of catalytic activity exhibited by the Chk1D469G protein, we wondered whether kinase activity was essential for its function. Therefore, we created a double mutant of the D469G allele with the D155A allele, a mutation in a highly conserved residue present in a multitude of protein kinases that compromises the catalytic activity of the protein [12]. As shown in Fig. 4B, mutation of D155A in the context of D469G completely inactivates the protein with regard to function as measured by the ability to confer survival upon exposure to UV light. Thus, while the catalytic activity of the D469G protein is minimal, it is nonetheless critical for the function of the D469G protein in conferring survival to a strain that otherwise lacks Chk1 function.

The kinase assay was originally designed to assay Chk1 activity toward a peptide substrate after a 10-minute incubation time. However, it is conceivable that the D469G protein may be activated at a later time period compared to the wild type protein and consequently is missed in the limited time frame of the assay. Therefore, we assayed activation of the mutant protein as compared to the wild type protein over a 45-minute time course. As with the single time point assay, WT Chk1 exhibits a basal level of kinase activity, which is stimulated 3-fold in response to damage as early as 5 minutes into the assay (Fig. 4C). In contrast, Chk1D469G exhibited a damage-induced increase in activity that remained far below the level of basal catalytic activity exhibited by WT Chk1 even after a 45-minute incubation time (Fig. 4C). As the low level of activity displayed by Chk1D469G demonstrably promotes checkpoint activation (Fig. 2A–B), while the Chk1D469A mutant does not, it was of interest to determine how the catalytic activity of the checkpoint deficient Chk1D469A mutant compared to D469G. As shown in Figure 4D, Chk1D469A possesses a basal level of kinase activity that falls below the range of basal activity displayed by Chk1D469G. Interestingly, when UV irradiated, Chk1D469A does undergo an increase in activity (Fig. 4D). Nevertheless, the level of UV-induced kinase activation in the Chk1D469A mutant fails to reach the levels of the damage-induced activation exhibited by the checkpoint proficient Chk1D469G and is clearly not adequate to promote checkpoint activation or cellular survival. Thus, it appears that Chk1D469G unveils threshold levels of phosphorylation and catalytic activity that are minimally required to trigger the DNA damage checkpoint. On the contrary, Chk1D469A with much lower catalytic activity and devoid of damage-dependent phosphorylation is incapable of checkpoint activation. These results suggest that Chk1D469G is partially functional while D469A represents a loss-of-function allele.

### Chk1D469G is checkpoint proficient for UV- or CPT-induced DNA damage

Cells expressing Chk1D469G display a level of survival to UV-irradiation that is comparable to that of a strain expressing WT Chk1, while cells expressing Chk1D469A are much more sensitive. To determine whether the difference in survival conferred by the two different alleles is the result of altered abilities to provide checkpoint function, we tested directly the ability of the mutant proteins to confer checkpoint function upon exposure to UV light or CPT.

**Figure 5:**
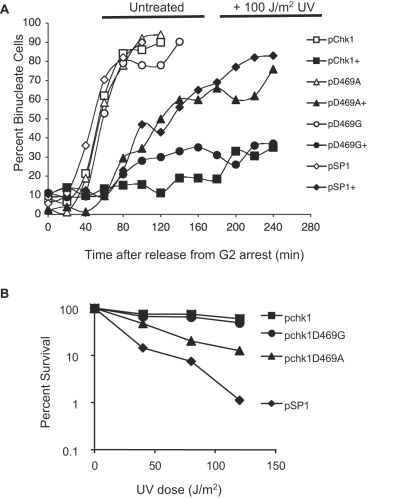
Chk1D469G, but not Chk1D469A, induces a cell cycle delay in response to UV treatment. A. The *chk1Δ cdc25-22* strains expressing the indicated plasmids were grown to mid-log phase at 25°C and shifted to 36.5°C for 2.5 hours to synchronize cell populations in G2. Cells were exposed to 100 J/m^2^ UV (untreated as a control) and released to 25°C to permit cycling. The percentage of binucleate cells was scored over the course of 4 hours. UV treatment groups are indicated by ‘+’ in the figure. B. The *chk1Δ* strain expressing the indicated plasmids were assayed for UV survival as described in Fig. 1B.

Checkpoint-proficient cells undergo a transient delay in mitotic entry upon exposure to damage [7]. The damage-dependent delay, and hence the integrity of the checkpoint, can be examined in a synchronous population of cells by monitoring the timing of mitotic entry following transient exposure to UV light or CPT [11]. As shown in Fig. 5A, this assay confirmed that the checkpoint is intact in UV-irradiated cells expressing Chk1D469G. While unirradiated strains begin to enter mitosis approximately 40 minutes after the release of the block, UV irradiation causes cells expressing wild-type Chk1 or Chk1D469G to delay mitotic entry. In contrast, cells devoid of Chk1 or that express Chk1D469A begin to enter mitosis at 80 minutes, with 40% of the cell population binucleate by 100 minutes. A direct comparison of the survival of cells expressing ChkD469G to cells expressing Chk1D469A reflects the checkpoint-proficient nature of the Chk1D469G cells (Fig. 5B). While the Chk1D469A mutant seems to be as checkpoint-deficient as cells with no Chk1 at all, they survive better than the *chk1* null strain. Reasons for this are unclear but likely reflect other roles for Chk1 in addition to activation of the checkpoint that may contribute to cell survival.

**Figure 6:**
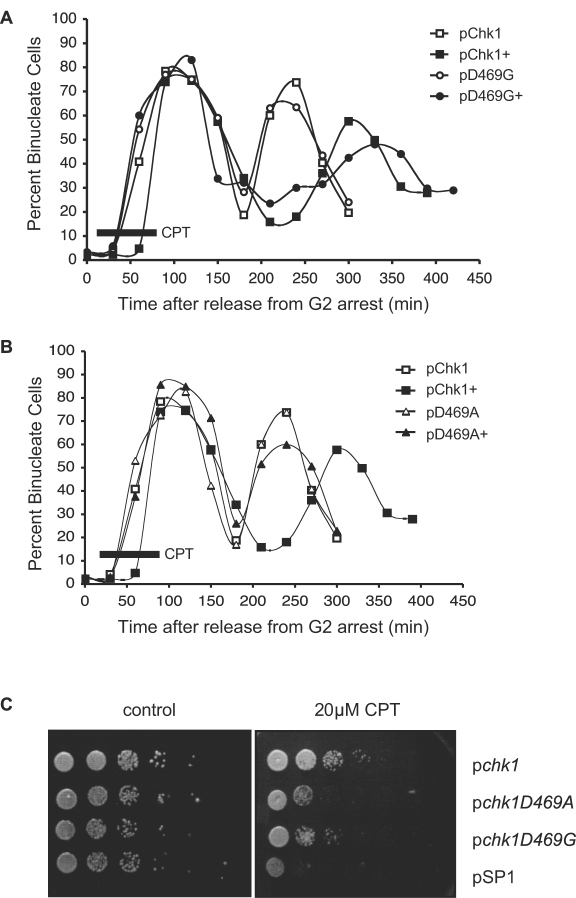
Chk1D469G, but not Chk1D469A, induces a cell cycle delay in response to CPT treatment. A. The *chk1Δ cdc25-22* strains expressing WT Chk1 or Chk1D469G were grown to mid-log phase at 25°C, shifted to 36.5°C for 2.5 hours to synchronize in G2, released to 25°C for 30 minutes, and treated with 20 µM CPT (untreated as a control). After a 1-hour incubation with CPT (time period indicated by black bar in figure and treatment groups indicated by ‘+’), cells were washed and maintained at 25°C for an additional 6 hours. The percentage of binucleate cells was scored over the course of 7.5 hours. B. The *chk1Δ cdc25-22* strains expressing WT Chk1 or Chk1D469A were examined as in A. C. The *chk1Δ* strains expressing the indicated plasmids were grown to mid-log phase, resuspended to 1×10^7^ cells/ ml and 10-fold serial dilutions were spotted on PMA plates containing 20 µM CPT (or no drug as a control). Plates were photographed after three days of growth at 30°C.

Since CPT induces topoisomerase I-mediated DNA strand breaks primarily during DNA synthesis in S phase [29], [30], the mitotic progression assay was modified in order to monitor the integrity of the checkpoint after CPT has poisoned topoisomerase I during S phase. Accordingly, cultures were synchronized in G2 and released for approximately 30 minutes to proceed through the first synchronous mitosis before treating with CPT in the subsequent S phase. The cells were subjected to 1 hour of CPT treatment, after which the drug was washed out to allow cells to recover and proceed to the second mitosis. Consequently, timing of mitotic entry was monitored over two periods of cell division. As shown in Fig. 6A, all strains proceed synchronously through the first mitosis. Entry into the next mitotic phase is delayed for approximately 60 minutes for cells expressing either wild-type Chk1 or Chk1D469G as a result of being challenged with CPT (Fig. 6A, compare closed to open symbols). When plotted, this mitotic delay manifests itself as a shift in the peak of percent binucleate cells from 240 minutes to 300 minutes.

In contrast, cells expressing Chk1D469A fail to delay mitotic entry in response to damage, as Chk1D469A cells exposed to CPT (Fig. 6B, closed triangles) proceed through mitosis with the same kinetics as the untreated strains (Fig. 6B, open symbols). Thus, the mutation of aspartic acid 469 to a glycine residue allows Chk1 to respond to UV irradiation and CPT treatment to maintain checkpoint function. On the contrary, an alanine substitution at this residue renders Chk1 defective and unable to execute a checkpoint-dependent delay when faced with UV or CPT-mediated DNA damage. As is the case for UV-induced damage, the checkpoint proficiency exhibited by the Chk1D469G allele translates into better survival for the strain in response to CPT treatment (Fig. 6C). On the other hand, and as was the case for UV damage, while the checkpoint deficiency exhibited by Chk1D469A correlates with poor survival, it does not render cells as sensitive to CPT as a *chk1* null strain (Fig. 6C).

### Chk1D469G is checkpoint deficient for some types of DNA damage

**Figure 7:**
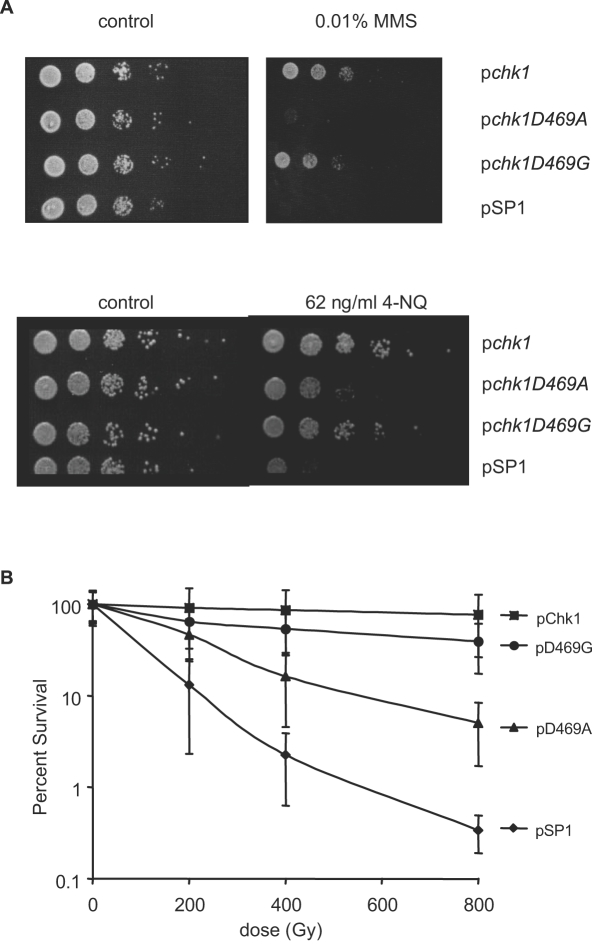
Chk1D469G, but not Chk1D469A, confers survival when cells are exposed to MMS, 4-NQ or gamma radiation. A. The *chk1Δ* strains prepared as in Fig. 6C were spotted on PMA plates containing 0.01% MMS (upper panel) or 62 ng/ml 4-NQ (lower panel). B. The *chk1Δ* strains transformed with the indicated plasmids were grown to mid-log phase, plated on PMA and irradiated with the indicated doses of gamma radiation from a ^60^Co source. Survival was calculated by determining colony number relative to unirradiated controls after 3 days of growth at 30°C.

It has been reported that the C-terminal region of Chk1 may activate the checkpoint in response to distinct DNA damage lesions [28], [31]. Therefore, to assess checkpoint functions of Chk1D469A and Chk1D469G we evaluated the ability of the mutant alleles to support survival upon generation of a variety of other types of DNA damage. As shown in Fig. 7A, cells expressing Chk1D469G survive exposure to an alkylating agent, MMS, or the UV-mimetic, 4-NQ. In contrast, cells expressing Chk1D469A exhibit sensitivity to these agents. We also examined sensitivity of these strains to double strand breaks generated by exposure of cells to gamma radiation. As shown in Fig. 7B, while cells expressing Chk1D469G are not as resistant as cells expressing wild-type Chk1, they are more resistant than cells expressing Chk1D469A or those that lack Chk1 altogether.

We also introduced these mutant alleles into strain backgrounds that would allow the generation of other DNA damage structures, specifically collapsed replication forks or unligated DNA. The *cds1* gene encodes a protein kinase with a primary role in S-phase [32]. Cds1 is essential for survival when DNA replication is inhibited by treatment with the inhibitor of ribonucleotide reductase, hydroxyurea (HU), presumably due to a role in stabilizing replication forks that have stalled as a result of depleted pools of ribonucleotide [32]–[34].

**Figure 8:**
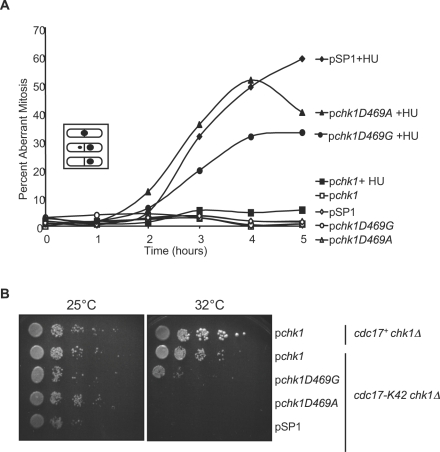
Chk1D469G cannot confer checkpoint function when replication forks stall or when DNA ligase activity is limited. A. A *cds1Δ chk1Δ* strain was transformed with the plasmids indicated in the legend. Mid-log phase cells were treated with 12 mM HU for the indicated period of time and monitored for the percentage of cells displaying aberrant mitosis. The key depicts cellular morphologies scored as aberrant mitosis. B. A *chk1Δ* strain transformed with WT Chk1 (*cdc17^+^*/pChk1) and *chk1Δ cdc17-K42* strains transformed with the indicated plasmids were grown to mid-log phase, resuspended to 1×10^7^ cells/ml and 10-fold serial dilutions were spotted on PMA plates. The plates were incubated at 25° and 32°C for 3 days.

Cells lacking Cds1 (*cds1Δ*) that have been exposed to HU exhibit activation of the DNA damage checkpoint as manifested by phosphorylation of Chk1 [34]. In the absence of Chk1, if *cds1Δ* cells are exposed to HU they undergo aberrant mitosis and display a “cut” phenotype, which is characterized by inappropriate nuclear division by the septa or uneven nuclear division leading to anucleate or aneuploid cells [32]. Strains expressing Chk1D469G or Chk1D469A display a high percentage of cells with the cut phenotype in the presence of 12 mM HU (Fig. 8A), while a *cds1Δ chk1Δ* strain expressing the wild-type *chk1* plasmid displays only 4.6% cut cells after 4 hours of HU treatment (Fig. 8A).

Limiting the activity of DNA ligase demands checkpoint activation for cell survival [7], [35]. The mutant form of DNA ligase encoded by *cdc17-K42* is functional at 25°C and completely non-functional at 36.5°C resulting in a block to cell cycle progression [36]. At an intermediate temperature of 32°C the Cdc17-K42 protein is partially functional prompting unligated Okazaki fragments to activate the checkpoint to induce a Chk1-dependent cell cycle delay [7], [35]. Consequently, a *chk1* deletion strain renders the *cdc17-K42* strain inviable at 32°C and can be exploited to test the checkpoint function of *chk1* alleles [26]. Expressing WT Chk1 allows colony formation at 32°C; however, like the empty vector control, expression of Chk1D469A mutant fails to allow colony formation and expression of Chk1D469G allows only minimal colony formation (Fig. 8B).

## Discussion

We have identified a Chk1 variant harboring a mutation in a highly conserved region of its C-terminus (Chk1D469G) that renders cells competent to withstand UV and CPT-induced DNA lesions, while an alanine substitution of the D469 residue (Chk1D469A) renders cells checkpoint deficient. The allele specificity of the UV/CPT resistance phenotype observed for Chk1D469G, coupled with the fact that the mutation lies within a region of the kinase that is highly conserved in evolutionarily distinct organisms, indicates that this C-terminal segment of Chk1 may be critical for detecting or responding to DNA damage signals.

We have previously shown that a prerequisite for a DNA damage-induced mitotic delay is phosphorylation of Chk1 on a critical C-terminal serine residue (S345) in a manner that requires the ATM/ATR-like kinase Rad3 [2]. The DNA damage-dependent phosphorylation of Chk1 has proven to be an adequate indication of checkpoint activation and is well-recognized in numerous model systems [37], [38]. Not only does Chk1 phosphorylation reflect checkpoint activation, but it also correlates well with robust survival following UV or CPT exposure [12], [26]. While Chk1D469G confers levels of survival that parallel the WT strain in response to either CPT or UV treatment, the level of phosphorylation of the mutant protein is much reduced as compared to wild type. Nonetheless, Chk1D469G is competent to induce a checkpoint dependent cell cycle delay. The ability of Chk1 mutants to function proficiently with minimal levels of phosphorylation is not unprecedented. Two independent groups observed that other C-terminal fission yeast mutants in the vicinity of D469G (E472D and I484T) exhibit significantly reduced phosphorylation levels and yet are checkpoint proficient [28], [31]. Interestingly, in an effort to elucidate the minimal structural requirements for checkpoint activation, *Xenopus* egg extracts were utilized to demonstrate that the length of single stranded DNA between the free ends of primed DNA, influenced the degree of Chk1 phosphorylation [39]. Perhaps the Chk1D469G protein interacts less efficiently with the damage signaling complex, thereby reducing the level of signaling to Chk1.

We have previously shown that Chk1 catalytic activity is crucial to elicit the DNA damage checkpoint response [12]. Evidence from mammalian cells, *Xenopus,* and fission yeast have established that Chk1 kinase activity is elevated in response to DNA damage signals, however, the precise mechanism of Chk1 activation has yet to be elucidated [12]–[14], [31], [40]. It has been hypothesized that the C-terminus of Chk1 acts to regulate its kinase activity by suppressing Chk1 catalytic activity until it is phosphorylated, at which point a conformational change leads to derepression of the catalytic domain. This conjecture is supported by both the crystal structure of the Chk1 catalytic domain and mutational studies that reveal C-terminal disruptions of Chk1 exhibit increased intrinsic kinase activity even in the absence of damage [21].

Bearing in mind these reports and their implication that the C-terminal domain of Chk1 may have a negative impact on its catalytic activity, we might have expected that the checkpoint proficient Chk1D469G mutant would display an elevated level of kinase activity both in the absence and presence of damage. However, we observed that Chk1D469G had significantly reduced basal activity that was stimulated greater than two-fold in response to damage and was apparently sufficient to enforce a mitotic delay. This implies that there is a threshold level of activity that is adequate to activate the checkpoint, and that WT Chk1 is normally activated in excess of the level that is minimally required to enforce a delay. Alternatively, it is possible that the absolute level of activity is not the critical factor for Chk1 function, but rather a change in magnitude of Chk1 activity may very well be the important parameter. It is noteworthy that a role for Chk1 in the spindle checkpoint has begun to emerge, requiring only the basal catalytic activity of Chk1 for function [41]. Phosphorylation of Chk1 in *Xenopus* in response to spindle perturbations does not affect the sites conventionally targeted by the ATM/ATR kinases. This suggests that either ATM/ATR have the potential to mediate phosphorylation of Chk1 at non-canonical sites, or alternatively, that phosphorylation is mediated by another caffeine-sensitive kinase.

Based on the substrate specificity determinants for Chk1 [24], we hypothesized that the region containing D469 might serve as a pseudosubstrate recognition site that mediates an autoinhibitory interaction with the catalytic domain. While it is quite possible that Chk1 is regulated by autoinhibition, we have shown here that mutations of the C-terminal residues I464 and R466 to alanine do not render Chk1 in a constitutively activated state. The I464A and R466A mutants retain function, though mutation of D469 to alanine inactivates the protein, both in terms of checkpoint function and catalytic activity. Intriguingly, mutation to glycine allows some function when Chk1D469G is expressed at greater than one copy per cell, though only when cells are challenged with some types of DNA damage. Glycine residues have a well-characterized architectural role in proteins as they often enable helical turns and bends (see for example [42]). In fact it has been suggested that glycine residues may be essential for switching proteins between distinct functional states because of the conformational freedom they provide to the protein backbone. We speculate that the glycine substitution creates a flexible hinge that permits Chk1 to exist in an open conformation. As a result, Chk1D469G is maintained in a functional state poised for mediating interactions with components of the checkpoint pathway albeit with reduced levels of phosphorylation and catalytic activity.

Alternatively, it is also possible that the Chk1D469G mutant is simply a partial, rather than complete, loss-of-function mutant. The ability of the D469G mutant to partially restore DNA damage-induced checkpoint function only when present in multiple copies supports this possibility, though it does not explain why Chk1D469G supports survival in response to some, but not all forms of DNA damage. To account for this observation, one might imagine that different types of DNA damage may demand different levels of Chk1 activity to promote survival. The reduced activity of Chk1D469G may be sufficient for lesions arising from base modifications or strand breaks, but not for conditions that generate collapsed forks or unligated DNA.

This is not the first report of lesion specific activation of fission yeast Chk1. Indeed, it has been reported that a particular set of *chk1* mutants can distinguish between lesions induced in G2 phase and structures generated by collapsed replication forks, and that these signal distinct checkpoint responses [28]. The mechanisms used by checkpoints to recognize different classes of DNA lesions are not clear. Some reports suggest that the ATM and ATR kinases target specific checkpoint components depending on the genomic lesion identified, while others suggest that it is not in fact lesion recognition, but the processing of diverse lesions by distinct DNA repair factors which ultimately recruits and activates particular checkpoint components [43], [44]. While the precise checkpoint activating structure remains elusive, studies in *Xenopus* suggest that ongoing replication may in fact alter the minimal requirements for checkpoint activation [39]. Thus, checkpoint activation during S phase likely differs from activation when replication has been completed.

Chk1 interacting proteins that promote damage specific responses have been identified. The BRCT motif-containing adaptor protein Crb2, which is required for Chk1 activation is required for checkpoint signaling induced by UV, methyl-methane sulfonate (MMS) and ionizing radiation, but is dispensable when cells are exposed to HU [6], [45]. However, we observe that supplying Crb2 in excess cannot rescue the survival of the single-copy checkpoint-deficient Chk1D469G mutant (unpublished data). In a Xenopus system, depletion of Claspin, a mediator protein for Chk1 activation, eliminates Chk1 phosphorylation in response to stalled replication forks, but depletion of both Claspin and BRCA1 is necessary to eliminate Chk1 phosphorylation when the damage signal arises from a double strand break [46]. By analogy, it is possible that Chk1D469G responds to some, but not all mediators of the DNA damage signal in S. pombe. We anticipate that an unbiased genetic screen to identify such factors that can support survival when Chk1D469G is present in single copy may reveal additional mediators that contribute to damage specific checkpoint responses.

A thorough understanding of the mechanism of Chk1-dependent checkpoint activation is critical for the development of anti-cancer strategies, as it is apparent that inactivating Chk1 can sensitize cells to genotoxic therapies [4], [5]. While loss-of-function alleles of Chk1 have been key to defining the role of Chk1 in the cellular response to DNA damage, it is clear that our understanding of the checkpoint response is far from complete. Mutant alleles of Chk1 provide valuable tools for genetically dissecting the checkpoint pathway and ultimately may contribute to the determination of how best to target Chk1 for therapeutic efficacy.

## Materials and Methods

### Plasmids, yeast strains, media, and growth conditions

Strains used in these studies are listed in Table 1. Mutations of *chk1* were constructed using as a PCR template the *S. pombe chk1* gene, containing 3 copies of the HA-epitope, cloned into the pSP1 multicopy plasmid [47]. Site-directed mutagenesis was performed using the Quick Change Site-Directed Mutagenesis Kit (Stratagene) to create the *chk1D363G, chk1D469G, chk1D469A, chk1I464A,*and *chk1R466A* point mutations. Mutations of the cDNA for *chk1* were also generated via site-directed mutagenesis, using as a template the HA-tagged *chk1* cDNA cloned into the pRep1 vector [48]. To confirm that only the desired base changes were established, the entire coding region of each *chk1* allele was sequenced. Subsequently, the plasmids were transformed using the LiAc transformation method as described into an *S. pombe* strain in which the endogenous copy of the *chk1* gene was disrupted with the *ura4* auxotrophic marker (*chk1::ura4* strain) [2]. *HindIII* or *EcoRV* fragments of genomic *chk1* alleles generated in pSP1 or pSP1-*chk1epΔHA*, respectively, were integrated into the *S. pombe* genome by gene replacement of the *chk1::ura4* allele as described previously for integration of the *chk1:ep* allele [2]. Southern blot analysis was performed to confirm integration of the mutant *chk1* alleles at the correct locus. Strains harboring plasmids were grown in Pombe Minimal (PM) medium made from Edinburgh Minimal Medium (Bio-101), prepared according to the manufacturers instructions and supplemented with adenine at 75 µg/ml (PMA). For the checkpoint assay, stains harboring the plasmid were grown in Synthetic Complete (SC) dropout medium supplemented with the appropriate amino acids at 75 µg/ml. Strains containing the alleles of *chk1* integrated into the genome were grown in rich yeast extract supplemented with adenine at 75 µg/ml (YEA) [49]. All strains were grown at 30°C, except in experiments utilizing temperature sensitive mutants. In experiments utilizing strains containing plasmids under the control of the *nmt1* thiamine repressible promoters, 20 µM thiamine was added to the medium to repress protein expression.

**Table 1:** Strains Used.

Strain	Genotype
972	*h^−^* (wild-type)
NW158	*h^+^ chk1::ura4 ura4-D18 leu1-32 ade6-216*
NW223	*h^+^ chk1:HA_3_ leu1-32 ade6-216*
NW554	*h^+^ chk1::ura4 ura4-D18 leu1-32 ade6-216/pREP1chk1:HA_3_*
NW569	*h^+^ chk1::ura4 ura4-D18 leu1-32 ade6-216/pREP1*
NW740	*h^+^ chk1::ura4 ura4-D18 leu1-32 ade6-216/pSP1chk1S345A:HA_3_*
NW1503	*h^+^ chk1::ura4 ura4-D18 leu1-32 ade6-216/pSP1chk1D469G:HA_3_*
NW1504	*h^+^ chk1::ura4 ura4-D18 leu1-32 ade6-216/pSP1:chk1D363G,D469G:HA_3_*
NW1505	*h^+^ chk1::ura4 ura4-D18 leu1-32 ade6-216/pSP1*
NW1506	*h^+^ chk1::ura4 ura4-D18 leu1-32 ade6-216/pSP1chk1:HA_3_*
NW1507	*h^+^ chk1::ura4 ura4-D18 leu1-32 ade6-216/pREP1chk1D469G:HA_3_*
NW1508	*h^+^ chk1::ura4 ura4-D18 leu1-32 ade6-216/pSP1chk1S345A,D469G:HA_3_*
NW1509	*h^+^ chk1D469G:HA_3_ leu1-32 ade6-216*
NW1510	*h^+^ rad3.T80 chk1::ura4 ura4-D18 leu1-32 ade6-216/pSP1chk1:HA_3_*
NW1511	*h^+^ rad3.T80 chk1::ura4 ura4-D18 leu1-32 ade6-216/pSP1chk1D469G:HA_3_*
NW1512	*h^+^ rad3.T80 chk1::ura4 ura4-D18 leu1-32 ade6-216/pSP1*
NW1513	*h^+^ chk1::ura4 ura4-D18 leu1-32 ade6-216/pSP1chk1D155A,D469G:HA_3_*
NW1514	*h^+^ chk1::ura4 ura4-D18 leu1-32 ade6-216/pSP1chk1D155A:HA_3_*
NW1515	*h^+^ chk1::ura4 ura4-D18 leu1-32 ade6-216/pSP1chk1D469A:HA_3_*
NW1516	*cdc2-3w cdc25:ura4 chk1::ura4 ura4-D18 leu1-32 ade6-216/pSP1chk1:HA_3_*
NW1517	*cdc2-3w cdc25:ura4 chk1::ura4 ura4-D18 leu1-32 ade6-216/pSP1chk1D469G:HA_3_*
NW1518	*cdc2-3w cdc25:ura4 chk1::ura4 ura4-D18 leu1-32 ade6-216/pSP1*
NW1519	*h^+^ cdc17-K42 chk1::ura4 ura4-D18 leu1-32 ade6-216/pSP1chk1:HA_3_*
NW1520	*h^+^ cdc17-K42 chk1::ura4 ura4-D18 leu1-32 ade6-216/pSP1chk1D469G:HA_3_*
NW1521	*h^+^ cdc17-K42 chk1::ura4 ura4-D18 leu1-32 ade6-216/pSP1*
NW1522	*cds1::ura4 chk1::ura4 ura4-D18 leu1-32 ade6-216/pSP1chk1:HA_3_*
NW1523	*cds1::ura4 chk1::ura4 ura4-D18 leu1-32 ade6-216/pSP1chk1D469G:HA_3_*
NW1524	*cds1::ura4 chk1::ura4 ura4-D18 leu1-32 ade6-216/pSP1*
NW1858	*h^+^ chk1::ura4 ura4-D18 leu1-32 ade6-216/pSP1chk1I464A:HA_3_*
NW1859	*h^+^ chk1::ura4 ura4-D18 leu1-32 ade6-216/pSP1chk1R466A:HA_3_*
NW1860	*h^+^ chk1::ura4 ura4-D18 leu1-32 ade6-216/pSP1chk1I464A,R466A:HA_3_*
NW1861	*h^+^ chk1::ura4 ura4-D18 leu1-32 ade6-216/pSP1chk1I464A,R466A,D469A:HA_3_*

### Genotoxic treatments

Camptothecin lactone (CPT) was obtained from the Drug Synthesis and Chemistry Branch, Developmental Therapeutics Program, Division of Cancer Treatment, National Cancer Institute. To analyze for Chk1 phosphorylation, exponentially growing cells were treated with 40 µM CPT for 2.5 hours as described [37]. To determine CPT sensitivity, strains were grown in liquid culture to mid-log phase, serially diluted ten-fold, and 5 µl aliquots were spotted on PMA plates containing 0, 15, and 20 µM CPT. To determine sensitivity to ionizing radiation, cells from log phase cultures were plated onto PMA plates at the appropriate concentration. The plates were exposed to ionizing radiation using a Cobalt^60^ source (Gammacell-220 Irradiator) for times calculated to deliver the indicated doses. Plates were incubated for 3–5 days at 30°C and colonies were counted. UV survival assays were performed as described [1]. To treat liquid cultures with UV, cells were collected on Durapore Membrane Filters (Millipore) via aspiration. The filters were UV irradiated and subsequently dropped into fresh media to release the irradiated cells from the filters. For treatment with hydroxyurea (HU), cells were incubated in the presence of 12 mM HU for up to 5 hours. Aliquots were taken at hourly intervals and fixed with 70% ethanol. To measure aberrant mitosis, the fixed cells were stained with Calcofluor to detect septa and 4,6-diamidino-2-phenylindole (DAPI) to visualize nuclei.

### Checkpoint Assays

Synchronous cultures were tested for the ability to arrest cell cycle progression in response to UV treatment as described [11] except that cells were propagated in SC-Leu drop out media to maintain the plasmids, and cells were fixed in ethanol for DAPI staining. For UV treatment, G2 arrested cells were collected on Durapore Millipore filters by aspiration, irradiated with 100 J/m^2^ UV, immediately washed off the filters into fresh SC-Leu media, and incubated at permissive temperature for 4 hours. To CPT treat synchronized cells, G2 arrested cells were released to permissive temperature for 30-minutes at which time they were treated with 20 µM CPT. Following 1 hour of treatment, the CPT was washed out, cultures were resuspended in fresh SC-Leu media, and maintained at permissive temperature for an additional 6 hours. Aliquots were collected every 30′, starting from the initial release to permissive temperature, and ethanol fixed for DAPI staining.

### Lysate preparation, immunoprecipitation, and immunoblotting

Cell lysates were extracted with IP buffer (10 mM NaPO_4_ pH 7, 0.15 M NaCl, 1% NP40, 10 mM EDTA, 50 mM NaF, 2 mM DTT, 50 mM PMSF, 50 mM Na-O-Va, and Complete Protease Inhibitors (Roche Diagnostics)). Cells were lysed using glass beads in a Fast Prep vortexing machine (Bio 101). The lysate was collected by centrifugation at 1000 RPM for 1 minute and spun for an additional 5 minutes at 5000 RPM to rid of cellular debris. SDS-PAGE and immunoblotting was performed as described [2].

For Chk1 immunoprecipitation, cells were lysed in IP buffer and 10 mg of the protein extracts was added to 100 µL of F7 anti-HA antibody (Santa Cruz). Each IP was brought up to a 500 µl volume and incubated at 4°C on a rotator for 1 hour, followed bythe incubation with 50 µl of protein A sepharose beads for 1 hour at 4°C. Subsequently, the IP's were washed three times in IP buffer and 120 µl of 2× Laemmli sample buffer was added to the dry beads. After a 5-minute boil, the IP's were split into 55 µl aliquots, run on 8% SDS-PAGE gels, and transferred to nitrocellulose for immunodetection with the Y11 anti-HA antibody at a 1∶1000 dilution (Santa Cruz). To detect phosphorylated Chk1, the blots were probed with the SN252-1 antibody, selective for *S. pombe* Chk1 phosphorylated on S345, at a dilution of 1∶1000 [12].

### Kinase Assay

Chk1 catalytic activity was assessed via *in vitro* kinase assays using 1–2 mg of protein per reaction as described [12]. For these kinase assays, Chk1 was immunoprecipitated with 40 µL F7 anti-HA antibody and 20 µL protein A sepharose beads.
